# Nrf2 Regulation by Curcumin: Molecular Aspects for Therapeutic Prospects

**DOI:** 10.3390/molecules27010167

**Published:** 2021-12-28

**Authors:** Seyed Hossein Shahcheraghi, Fateme Salemi, Niloufar Peirovi, Jamshid Ayatollahi, Waqas Alam, Haroon Khan, Luciano Saso

**Affiliations:** 1 Infectious Diseases Research Center, Shahid Sadoughi Hospital, Shahid Sadoughi University of Medical Sciences, Yazd 8916978477, Iran; shahcheraghih@gmail.com (S.H.S.); jamshidayatollahi@yahoo.com (J.A.); 2 School of Medicine, Islamic Azad University of Medical Sciences, Yazd 19395/1495, Iran; fatemesalemi77@gmail.com; 3 School of Medicine, Tehran University of Medical Sciences, Tehran 1417614411, Iran; niloufarpeirovi@gmail.com; 4 Department of Pharmacy, Abdul Wali Khan University Mardan, Mardan 23200, Pakistan; waqasalamyousafzai@gmail.com; 5 Department of Physiology and Pharmacology “Vittorio Erspamer”, Sapienza University, 00185 Rome, Italy; luciano.saso@uniroma1.it

**Keywords:** curcumin, Nrf2 regulation, mechanistic insight, anticancer effect, future prospect

## Abstract

Nuclear factor erythroid 2 p45-related factor (2Nrf2) is an essential leucine zipper protein (bZIP) that is primarily located in the cytoplasm under physiological conditions. Nrf2 principally modulates endogenous defense in response to oxidative stress in the brain.In this regard, Nrf2 translocates into the nucleus and heterodimerizes with the tiny Maf or Jun proteins. It then attaches to certain DNA locations in the nucleus, such as electrophile response elements (EpRE) or antioxidant response elements (ARE), to start the transcription of cytoprotective genes. Many neoplasms have been shown to have over activated Nrf2, strongly suggesting that it is responsible for tumors with a poor prognosis. Exactly like curcumin, Zinc–curcumin Zn (II)–curc compound has been shown to induce Nrf2 activation. In the cancer cell lines analyzed, Zinc–curcumin Zn (II)–curc compound can also display anticancer effects via diverse molecular mechanisms, including markedly increasing heme oxygenase-1 (HO-1) p62/SQSTM1 and the Nrf2 protein levels along with its targets. It also strikingly decreases the levels of Nrf2 inhibitor, Kelch-like ECH-associated protein 1 (Keap1) protein.As a result, the crosstalk between p62/SQSTM1 and Nrf2 could be used to improve cancer patient response to treatments. The interconnected anti-inflammatory and antioxidative properties of curcumin resulted from its modulatory effects on Nrf2 signaling pathway have been shown to improve insulin resistance. Curcumin exerts its anti-inflammatory impact through suppressing metabolic reactions and proteins such as Keap1 that provoke inflammation and oxidation. A rational amount of curcumin-activated antioxidant Nrf2 HO-1 and Nrf2-Keap1 pathways and upregulated the modifier subunit of glutamate-cysteine ligase involved in the production of the intracellular antioxidant glutathione. Enhanced expression of glutamate-cysteine ligase, a modifier subunit (GLCM), inhibited transcription of glutamate-cysteine ligase, a catalytic subunit (GCLC). A variety of in vivo, in vitro and clinical studies has been done so far to confirm the protective role of curcumin via Nrf2 regulation. This manuscript is designed to provide a comprehensive review on the molecular aspects of curcumin and its derivatives/analogs via regulation of Nrf2 regulation.

## 1. Introduction

As a major monomer constituent in turmeric derived from *Curcuma Longa* rhizomes, curcumin has shown a variety of potential bioactivities such as anti-inflammatory, antioxidant and anti-infective properties, with few side effects [1,2,3,4,5,6,7]. To a great extent, there are numerous chemical and natural activators of nuclear factor erythroid 2-related factor 2 (Nrf2), and curcumin is considered as one of these biological activators. Nrf2 is a basic leucine zipper protein (bZIP) that is mainly located in the cytoplasm under physiological conditions. In response to oxidative stress in the brain, Nrf2 primarily regulates endogenous defense. In that regard, Nrf2 translocates into the nucleus and heterodimerizes with the small Maf or Jun proteins. Afterwards, it binds to specific DNA sites such as antioxidant response elements (ARE) or electrophile response elements (EpRE) to initiate the transcription of cytoprotective genes in the nucleus [8,9,10].

Mainly examined clinical studies have limited success, primarily because of limited bioavailability and rapid metabolism. In these studies, enone Analogues of curcumin examined to identify Nrf2 activators were divided into the following groups:enone with a 7-carbon dienone spacer, 5-carbon enone spacer (with and without a ring) and a 3-carbon enone spacer. As a result, there were activators of Nrf2 in all groups, many of which were more active than curcumin. Dose–response studies suggest that these analogs activate Nrf2 by multiple mechanisms, including the sensitivity to activation, reflected in EC_50_ values and the extent of activation influenced by a broad range of substituent on the aromatic rings of these enones [11,12,13,14,15,16,17,18,19,20,21,22,23,24,25].

Since these studies show curcumin conspicuously elevates the levels of nuclear expression and also by interaction with Cys151 in Keap1 increases the biological effects of Nrf2. Therefore, curcumin could be a prodigious therapeutic choice for various oxidative stress-related diseases, including type 2 diabetes mellitus, neurodegenerative diseases, cardiovascular diseases, malignancies and viral infections such as even more recent ones SARS-CoV-2 [26,27,28,29].

The majority of PEGylated curcumin analogs vigorously promoted Nrf2 function compared with free curcumin. Copolymer 3a was identified as the most potentcomponent in PEGylated curcumin, strengthening Nrf2-dependent NQO1 production in a dose-dependent pattern [30].

## 2. Curcumin and Elevated Nrf2 in Preventing Cancer

The transcription factor Nrf2’s major role is to stimulate the antioxidant defense responses via promoting the transcription of numerous genes in order to shield cells against endogenous and exogenous stressors like oxidative stress and xenobiotic compounds. Therefore, as a consequence, Nrf2 is generally interpreted as a cytoprotective transcription factor that serves as the cell’s primary protection mechanism and a key modulator of cellular function. Constant detection of hyper activated Nrf2 in many neoplasmsstrongly suggesting its responsibility for cancers with poor prognosis. Exactly like Curcumin, Zinc–curcumin Zn (II)–curc compound has been shown to induce Nrf2 activation. In the cancer cell lines analyzed, Zinc–curcumin Zn (II)–curc compound can also display anticancer effects via diverse molecular mechanisms, including markedly increasing heme oxygenase-1 (HO-1) p62/SQSTM1and the Nrf2 protein levels along with its targets. It also strikingly decreases the levels of Nrf2 inhibitor, Keap1 protein.Therefore, the crosstalk between p62/SQSTM1 and Nrf2 could be therapeutically exploited to increase cancer patient response to therapies [31].

Inhibition of carcinogenesis may be a consequence of attenuation of oxidative stress via activation of the antioxidant defense system, restoration and stabilization of tumor suppressor proteins and modulation of inflammatory mediators. As previously have delineated, Curcumin, during its long-term effect, has a significant role in regulating Nrf2 mediated phase-II antioxidant enzymes and the tumor suppressor p53. In addition, Nrf2 signaling inhibition, downregulated the phase II antioxidant enzymes like GST, GR, NQO1 and tumor suppressor p53 level. It activated the inflammatory signals via upregulation of TGF-β and reciprocal regulation of iNOS and COX2 in the liver of lymphoma-bearing mice [32,33].

Although the mechanism of Curcumin’s inhibition of breast cancer cell proliferation is not yet fully understood, underlying mechanisms have been demonstrated. Fen1 is a DNA repair-specific nuclease that its Over-expression is involved in the development of breast cancer. Curcumin, while inhibiting Fen1 protein expression, remarkably down-regulated the Nrf2 protein expression. It could also inhibit the Fen1-dependent proliferation of MCF-7 cells. Further investigation revealed that curcumin could lead to reduce Fen1 gene expression in an Nrf2-dependent manner. Nrf2 translocation from the cytoplasm to the nucleus and induce the activity of Fen1 promoter [34].

**Figure 1:**
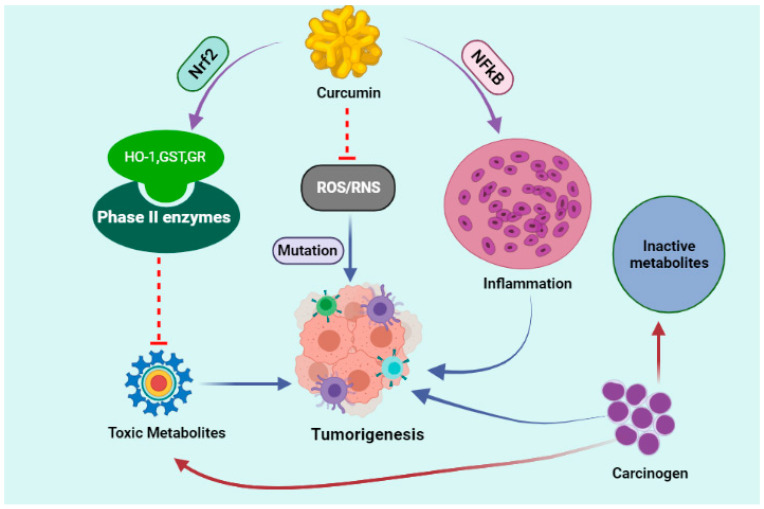
Anti-tumorigenesis effect of curcumin via targeting different pathways including inhibition of ROS (reactive oxygen species); RNS (reactive nitrogen species); NFкB (nuclear factor kappa B); Nrf2 (NF-E2-related factor 2); HO-1(heme oxygenase-1); GST (glutathione S-transferase); GR (glutathione reductase).

Amino-1-methyl-6-phenylimidazo [4,5-b]pyridine(PhIP) is a food carcinogen and could be found in cooked meat. As PhIP metabolites generate DNA adduct and DNA strand breaks it can cause multiple types of malignancies such as breast cancer. To date, we know, curcumin crucially inhibited the formation of DNA adduct induced by PhIP (Figure 1) and with attendant decreased production of reactive oxygen species (ROS), DNA double strand breaks. Nrf2 expresses and FOXO targets; DNA repair genes BRCA-1, H2AFX and PARP-1. PhIP reduced the expression of the tumor suppressor P16 gene, whereas co-treatment of curcumin increased this expression. Caspase-3 and -9 were slightly suppressed by curcumin with consequent inhibition of cell death. These results suggest that curcumin appears to be an effective anti-PhIP food additive, likely acting through multiple molecular targets [35].

One of the most lethal men malignancies in the United States of America is Prostate cancer (PCa). Although PCa could localize effectively and be treated by surgery or radiation, usually metastatic PCa has a poor prognosis.Recently in TRAMP mice demonstrated that Nrf2 was epigenetically silenced through the tumorigenesis of the prostate. Using bisulfite genomic sequencing (BGS) showed that curcumin reversed the methylation status of the first 5 CpGs in the promoter region of the Nrf2 gene and could also remarkably suppress the anti-mecyt antibody binding to the first 5 CpGs of the Nrf2 promoter.Taken together, at least some part of curcumin potentially could have a preventive effect in prostate cancer via suppression of the Nrf2-mediated antioxidative stress cellular defense pathway [36,37].

## 3. Nrf2 Is an Essential Factor for Curcumin Effects on the Cellular Processes and Enzymes

### 3.1. In Vitro Studies

#### 3.1.1. Renal Epithelial Cells

Oxidation–reduction reactions stimulate the inactivated form of transcription factor Nrf2 bound to Keap1 protein in the cytoskeleton by altering their interactions. The activated Nrf2 is transported to the nucleus, a process known as nuclear translocation, and attaches to AREs to stimulate the expression of ARE genes. AREs promote the production of proteins such as hem oxygenase-1 (HO-1) that secure cellular function against inflammatory and oxidative factors. Moreover, negatively charged molecules such as polyphenols and herbal derivatives can trigger Nrf2-Keap1 pathway. Recent studies focus on the mechanisms by which curcumin and caffeic acid phenethyl ester indirectly (CAPE) stimulates HO-1 via Nrf2 activation in renal epithelial cells. In addition, curcumin and CAPE facilitate Nrf2 and HO-1AREs interactions in a dose and time-dependent pattern [38,39,40].

#### 3.1.2. Lung Mesenchymal Stem Cells

The buildup of ROS within lung mesenchymal stem cells (LMSCs) interferes with their function and survivalin idiopathic pulmonary fibrosis. Keet al. assessed the antioxidative mechanisms of curcumin againsthydrogen peroxide (H_2_O_2_) induced oxidative stress in murine LMSCs. The results showed a dose-dependent reduction in apoptosis rate and reactive oxygen specieslevels. Curcumin enhanced redox reactions by intensifying mitochondrial membrane capacity. In addition, it is associated with decreased cleaved caspase-3 production, Nrf2 dependent HO-1 activation and antiapoptotic features through augmentation of p-Akt/Akt and Bcl-2/Bax ratios [41]. In addition, curcumin enhanced the maturation of human periodontal ligament stem cells curcumin promotes the by promoting PI3K/AKT/Nrf2 signaling cascade [42].

The oxidized surfactant in heights with low oxygen saturationleads to increased alveolar surface tension and alveolar collapse.Hypoxic-induced oxidative stress resulted in a catabolic state by oxidizing lipids and proteins and decreasing the antioxidative Nrf2 HO-1 pathway. To investigate the antioxidative characteristics of curcumin, it was used as prevention before induction of hypoxia in A549 cells and exposure of male Sprague–Dawley rats tohigh hypoxic altitudes. Curcumin elevated phase II antioxidants levels by activating BALF and hypoxia inducible factor 1(HIF-1α) and inhibiting vascular endothelial growth factor (VEGF). Curcumin increased surfactant production and prevented degradation, therefore, revived oxidative and antioxidative balance [43]. Lung cancer cells’ lack of response to chemotherapy such ascisplatin (CDDP)-based regimen might be attributed to impaired Nrf2 function. Since human lung’s adenocarcinoma (A549/CDDP) cells, that received CDDP triggered autophagy and anomalous Nrf2 induction. However, curcumin implication maintained Nrf2-Keap1 connection and inhibited vigorous Nrf2 activation [44].

#### 3.1.3. The Role of Nrf2-Keap1 Signaling Pathway in Protecting Macrophages against Oxidative Stress

**Figure 2:**
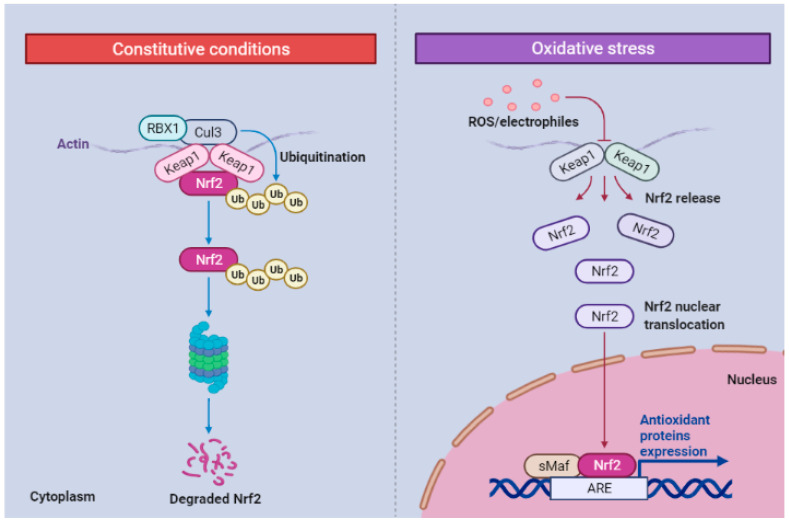
Schematic representation ofNrf2regulation viaKEAP1 Pathway.Nrf2 is consistently ubiquitinated by KEAP1 and destroyed in the cytosol proteasome at normal conditions. During stress, the KEAP1-Nrf2 connection is disrupted, allowing free NRF2 to translocate into the nucleus. Nrf2 then forms heterodimers with sMaf and binds to ARE sites in antioxidant and detoxification genes’ regulatory regions. Nrf2;(nuclearerythroid2likefactor-2); KEAP1(Kelch-likeECH-associatedprotein1); AR (antioxidant response element);sMafs;(small musculoaponeuroticfibrosarcoma oncogene family).

As one of the major elements of the immune system, macrophages require potent antioxidant factors to survive in microenvironments with expanding amounts of ROS. Lin et al. selected RAW264.7 cells and imposed oxidative load via hydrogen peroxide (H_2_O_2_) in cells that received curcumin, redox enzymes including catalase (CAT), superoxide dismutase (SOD) and glutathione peroxidase (GSHPX), functioned more effectively.Despite low- and intermediate- dosage, higher amounts of curcumin elevated intracellular MDA and ROS. Low-dose curcumin demonstrated antiapoptotic features while increasing curcumin amounts failed to defend the cells against apoptosis during the initial stages of development. A moderate dosage of curcumin-activated antioxidant Nrf2 HO-1 and Nrf2-Keap1 pathways (Figure 2) and upregulated the modifier subunit of glutamate-cysteine ligase involved in the production of the intracellular antioxidant glutathione. Enhanced expression of glutamate-cysteine ligase, a modifier subunit (GLCM), but inhibited transcription of glutamate-cysteine ligase, a catalytic subunit (GCLC) [45].

#### 3.1.4. Lps-Induced Macrophage Damage

LPS up-regulated cytokines involved in inflammation such as iNOS, interleukin-6 (IL-6) and TNF-α. Therefore, Boyanapalli et al., evaluated the effects of phenethyl isothiocyanate (PEITC) and curcumin on antioxidative and anti-inflammatory features of the Nrf2 pathway in LPS-induced peritoneal macrophage damage. DespiteNrf2+/+ macrophages, PEITC and curcumin failed to increase the expression of anti-inflammatory mediators and HO-1 in Nrf2 knockout macrophages. However, these phytochemicals inhibited COX-2 production involved in inflammatory reactions inNrf2−/− macrophages [46].

Dysregulated fatty acid metabolism causesfatty acid-induced hepatic damage in Non-alcoholic fatty liver disease (NAFLD). Yan et al. investigated curcumin interactions with bile acid and xenobiotics metabolic reactions in C57BL/6 mice with NAFLD. It seemed that curcumin decreased hepatocellular fat storage, improved liver function profile and boosted the metabolic function of CYP3A and CYP7Ain mice with fatty liver.The regulatory effect of curcumin on fatty acid synthesis was evidenced by a decrease in fatty acid metabolic components including CD36, SREBP-1c, LXR-α and FAS.However, the enhanced FXR, SHP and Nrf2 pathways in the C57BL/6 cell line seemed to synergistically increase the antioxidative capacity of each pathway [47].

#### 3.1.5. Curcumin against Hepatotoxic Effects of Quinocetone (QCT)

QCT adversely affects cellular survival by promoting oxidative enzymes such as lactate dehydrogenase (LDH) and apoptosis. Dai et al. found that applying curcumin before QCT inL02 liver cells relieving oxidative stress improved Nrf2/HO-1activity and mitochondrial redox reactions. Moreover, the boosting impact of QCT onNF-kB signaling cascade and inducible nitric oxide synthase (iNOS) function in NO synthesis was constricted by curcumin in vitro [48].

#### 3.1.6. Arsenic Toxicity

Arsenic disturbed intracellular oxidative and antioxidative balance by reducing glutathione and promoting LDH function, leading to genome destruction, decreased survival rate and consequently apoptosis. It restrained intracellular antiapoptotic components such as mTOR, Akt, Nrf2, ERK1, Bcl-x and Xiap and supported cell death via promoting apoptotic factors; ULK, LC3, p53, Bax, cytochrome c, caspase 9, cleaved caspase 3 proteins.However, curcumin defended the PC12 cell line from every cytotoxic effect mainly through Nrf2 up-regulation [49,50].

Inorganic arsenic causes malignant and non-malignant skin pathologies. Curcumin induced the Nrf2 pathway, a dose and time-dependent pattern that defended human HaCaT keratinocytes from oxidative stress. However, high curcumin boosted NRF1, the lower doses down-regulated apoptoticenzymes such as cleaved caspase-3 and cleaved PARP protein and protected HaCaT cells death from arsenic cytotoxicity. The antioxidative effects of curcumin were limited due to Nrf2 or *KEAP1* deactivation, proving the dependence of curcumin’s antioxidative features on these signaling pathways [51].

Aldose reductase (AR) protects cells from the oxidative stress of toxic aldehydes. Curcumin enhanced Nrf2 and phosphatidylinositol 3-kinase/Akt pathways to increase the expression of AR. This effect is dependent on the presence of active NFkB since curcumin failed to assert its Nrf2 related antioxidative properties in the setting of NFkB inhibition [52,53].

#### 3.1.7. Cytotoxic Effects of Zearalenone (ZEA) on Leydig Cells

Curcumin prophylaxis improved oxidative load and cellular death in Leydig cells that underwent zearalenone (ZEA) by activatingPI3K-AKT and Nrf2 pathway that prevents apoptosis [54]. Production of ROS inplacenta trophoblasts is regarded as one of the pregnancy-associated side effects. Curcumin pretreatment revealed human trophoblasts HTR8/SVneo cells from H_2_O_2_ induced oxidative stress and apoptosis through constraining cleaved-caspase 3.It also increased antioxidative enzymes by inducing Nrf2 signaling and subsequent elevation of Bcl-2/Bax ratio [55].

In osteoblasts impaired by elevated ROS levels, curcumin decreased oxidative stress and supported osteoblasts’ survival and reproduction by suppressing Glycogen synthase kinase (GSK3b) and reinforcing Nrf2. Therefore, further investigations are required to clarify curcumin’s antiosteoporotic features [56].High glucose in diabetic retinopathy exposes human retinal pigment epithelial (RPE) cells to oxidative injury. Curcumin promoted Nrf2/HO-1 and ERK1/2 expression and inhibited ROS-induced apoptosis in RPE cells [57,58].

#### 3.1.8. Curcumin Potential Therapeutic Applications against AMD (Age-Related Macular Degeneration)

Despite the low bioavailability of curcumin, its analog 1, 5-bis (2-trifluoromethylphenyl)-1, 4-pentadien-3-one (C3), displayed more antineoplastic and anti-inflammatory capacities compared with curcumin. In the human retinal pigment epithelial cell line (ARPE-19), C3 impeded cytotoxic effects of acrolein via direct activation of the Nrf2 signaling cascade. It boosted mitochondrial reductive enzymes and GSH and defended ARPE-19 cells against oxidative stress more vigorously than curcumin [59].

In Fuchs endothelial corneal dystrophy, the endothelium increased ROS load is indicated for a corneal transplant. Curcumin was used to avoid oxidative stress in corneal endothelial cells (CECs) to see whether it could defend CECs. It was found that curcumin exerts its cytoprotective effects through activation of Keap1/Nrf2/ARE pathway [60]. The association of 1,25D/CA with 1,25D curcumin failed to increase Nrf2 signaling in mononuclear neoplastic cells of chronic myelogenous leukemia (CML) and acute myeloid leukemia (AML). NQO1 gene was only expressed in AML cells that received either or both of these antioxidants [61].

One of the proapoptotic features of P53 is to prevent intracellular antioxidative pathways such as Nrf2. In neoplastic cells with impaired P53 function, the Nrf2 path is one of the significant culprits of the immortality of tumor cells in P53 impaired neoplastic cells. Early curcumin exposure upregulated apoptotic pathways DNA fragmentation, phosphatidylserine exposure and caspase-3, caspase-9 and poly (ADP-ribose) polymerases (PARP) cleavage) by enhancing Nrf2 nuclear translocation in P53 knockout lymphoblastoid cells.As time passed, late doses of this polyphenol deactivated Nrf2 antiapoptotic characteristic. Therefore, it can be concluded that curcumin downregulation of Nrf2 promoted cell death independent ofP53 proapoptotic regulations [62].

**Figure 3:**
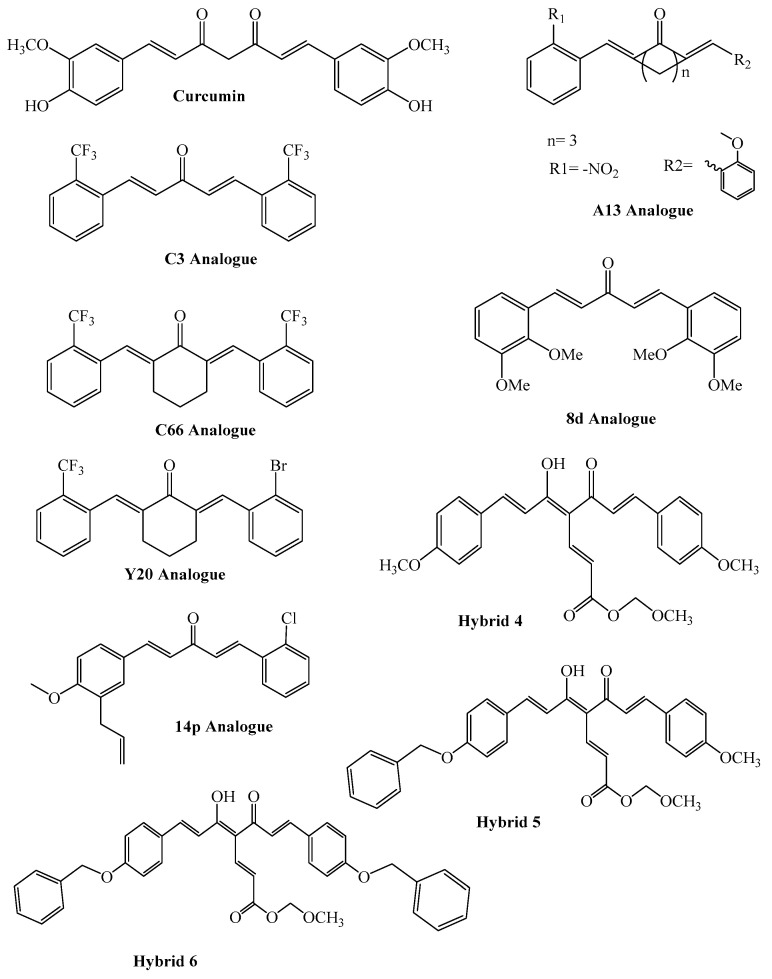
Structures of Curcumin and its different analogues.

Human glutathione S-transferaseP1 (GSTP1) is another cellular antioxidant involved in the detoxification of hepatocytes from cytotoxic effects of drugs and other toxins. Curcumin applied cytoprotective features against ROS and cancer development in HepG2 cellsthrough activation of antioxidant response element (ARE) segment of human GSTP1 [63]. Chemical structures of curcumin and its different analogues have been shown in Figure 3.

### 3.2. Animal Studies

#### 3.2.1. How Curcumin Inhibits Renal Cell Damage in Rats Undergoing 5/6 Nephrectomy

The kidney is adversely affected by renal excision since 5/6 nephrectomy (5/6NX) procedure imposes oxidative stress, increased glomerular filtration rate (GFR) and hypertensive nephropathy. These side effects are primarily due to glomerular impairment and downregulation of Nrf2-Keap1 signaling pathway. Curcumin has shown inhibitory effects against renal cell damage. By promoting the Nrf2 pathway, this natural monomer acts against destructive oxidative enzymes that lead to renal failure. Tapia et al., investigated the role of curcumin in protecting rat renal cells against nephrectomy side effects. They found that implication of curcumin a week before 5/6NX improved renal function by decreasing hypertension, protein excretion, glomerulosclerosis, interstitial fibrosis, inflammation, blood urea nitrogen (BUN) and creatinine. Curcumin exerts its protective effects by activating Nrf2, which is involved in the expression of enzymes that attenuate oxidative pathways [64].

The combination of Thymoquinone (Tq) and curcumin demonstrate synergistic interaction against cisplatin-induced nephrotoxicity in rats. Impaired renal cells by this chemotherapy drug showed decreased GFR, expression ofinflammatory mediators including TNF-α, IL-6, MRP-1, elevated serum BUN, creatinine and CK levels. Thymoquinone (Tq) and curcumin act against these adverse effects and prevent apoptotic and antiproliferative effects of cisplatin on HEK-293 cell lines. This study found that oxidation-reduction enzymes and ATP synthases were protected from cisplatin destructive impacts caused by KIM-1pathway in rats that received Thymoquinone (Tq) and curcumin intervention [65].

#### 3.2.2. Lipopolysaccharide (LPS)/D-Galactosamine (D-GalN)-Induced Acute Liver Injury (ALI)

ROS plays a significant role in the pathophysiology of liver dysfunction by promoting inflammatory and apoptotic microenvironments. Due to LPS, curcumin decreased inflammatory chemokines such as Interleukin 1beta (IL-1β), Interleukin 6 (IL-6) and TNF-a in mice with ALI.Furthermore, curcumin inhibited apoptotic signaling cascades such as mitogen-activated protein kinases/c-Jun NH2-terminal kinase (P38/JNK) and phosphatidylinositol 3-kinase/protein kinase B (PI3K/AKT). It reduced the expression of significant factors associated with oxidative stress, including Cyclic AMP-responsive element-binding protein (CREB)/Caspase and oxidative stress-associated protein. Curcumin also enhanced the expression of antioxidant enzymes like superoxide dismutase (SOD), catalase (CAT), glutathione (GSH) and glutathione peroxidase (GSH-px) in rat hepatocytes [66].

In rat hepatic stellate cells (HSCs), glucose oxidase (GO) increased the production of ROS, MDA, muscle α-actin (α-SMA), extracellular matrix (ECM) and glutathione (GSH) to a lower degree. Nevertheless, curcumin efficiently reduced oxidative load via reducing ROS, MDA, α-SMA and ECM while enhancing Nrf2 and GSH [67,68].

Aflatoxins (AF), especially Aflatoxin B1 (AFB1), an Aspergillus flavus, and Aspergillus parasiticus derivative, have shown mutagenic and carcinogenic features in mammals and poultry by causing multiorgan failure. Despite the well-known supporting effects of curcumin on Nrf2, little is understood about its impacts on downstream genes such as glutathione (GST) isoforms that play a crucial part in protecting Arbor Acres broilerhepatocytes against the destructive effects of AFB1 [69,70]. Wang et al. investigated the impacts of curcumin-activated Nrf2 on GST subtypes in Arbor Acres broiler hepatocytes. The histopathologic assessment showed that curcumin prevented hepatic injury in AFB1 exposed liver cells with a dose-dependent pattern. It contained inhibitory effects of AFB1 on Nrf2 antioxidant properties, enhancing the expression of phase-II enzymes by supporting AFB1-GSH conjugation [71].

Applying curcumin before induction of ALI prevented hepatocellular dysfunction and inflammation, which can be concluded from lower ALT and AST levels and decreased intracellular MDA in rats. Moreover, inflammation caused by TNF-α and upregulated NF-kB pathway was improved. Curcumin enhanced the expression of HO-1, glutamate-cysteine ligase GCLC, NAD(P)H dehydrogenase and quinone (NQO-1) in a dose-dependent pattern [72,73,74].

#### 3.2.3. Hepatotoxicity

##### Arsenic

The Nrf2 signaling pathway and associated antioxidative enzymes can defend hepatocytes against oxidative stress due to inorganic arsenic intoxication. The cell protection capacities of curcumin in ROS increment are evident in vivo studies. It avoids the accumulation of malonaldehyde (MDA) in liver cells and decreases blood levels of hepatic glutathione (GSH), alanine aminotransferase (ALT) and aspartate aminotransferase (AST). Curcumin also facilitates renal arsenic excretion by enhancing its methylation. Additionally, NADP(H) quinine oxidoreductase 1 (NQO1) and HO-1 as subsequent signaling cascades of Nrf2 were activated [75].

##### Alcohol

Curcumin stimulates Nrf2/p53 signaling to diminish ethanol-induced hepatotoxicity and apoptosis, which may be a potential therapeutic option for alcoholic liver disease (ALD) [76,77,78,79,80,81]. According to a recent study that evaluated the antioxidative characteristics of curcumin in high temperatures, this polyphenol attenuates oxidative stress in birds affected by heat via a dose-dependent decrease in heat shock protein 70 [82,83].

#### 3.2.4. Anti-Inflammatory Aspects of Curcumin in Close Association with Its Antioxidative Features

The interconnected anti-inflammatory and antioxidative properties of curcumin resulted from its modulatory effects on Nrf2 signaling pathway have been shown to improve insulin resistance. Curcumin exerts its anti-inflammatory impacts through suppressing metabolic reactions and proteins such as Keap1that provoke inflammation and oxidation. Moreover, the role of inflammatory mediators like tumor necrosis alpha (TNF-α)in supportingKeap1 production andNrf2 pathway inactivation is prevented mainly by this natural polyphenol [84].

#### 3.2.5. Curcumin Can Act against the Progression of Cardiovascular Disorders

Fatty acids play a significant role in the pathophysiology of cardiovascular disorders. An in vitro and in vivo study elucidated curcumin’s antioxidative and anti-inflammatory impacts in protecting cardiac-derived H9C2 cells. Curcumin efficiently attenuated oxidative stress, decreased cellular survival and pro-inflammatory injuries caused by palmitate. Furthermore, oral curcumin 50 mg/kg improved the cardiotoxic effects of a fat-rich diet in mice. Curcumin employed its antioxidative, antiapoptotic and anti-inflammatory effects through activating Nrf2 and inhibiting NF-κB signaling pathways. Therefore, these cascades may act as potential therapeutic targets against the cardiotoxic effects of free fatty acids [85].

Obesity and hyperlipidemia are associated with an increased risk of cardiovascular disorders due to elevated ROS and inflammatory mediators. Qian et al. assessed the efficiency of a structurally modified curcumin called Y20 on rats that received high amounts of dietary fat. According to the results, 20 mg/kg Y20, equivalent to 50 mg/kg curcumin, protected cardiac cells from remodeling, hypertrophy, fibrosis and apoptosis by promoting Nrf2 and impeding NF-κB functions [86].

#### 3.2.6. Curcumin Reinforced Cardioprotective Impacts of Metformin in Diabetic Rats

Diabetes caused myocardial dysfunction and elevated cardiac enzymes such as creatine kinase-MB (CK-MB) and troponin I. In addition, a considerable increase in inflammatory mediators including TGF-β1, IL-6 and NF-κB as well as oxidized lipids was noted. However, histologic assessments demonstrated signs of cardiac injury in the absence of antioxidants in rats that underwent metformin therapy. On the other hand, the combination of metformin and curcumin therapy appeared to defend cardiac cells against oxidative stress through JAK/STAT and Nrf2/HO-1 pathways [87,88].

The increased load of ROS plays a significant role in diabetic-induced cardiomyopathy. A13, another curcumin analog, is known to be more potent than the cumin itself. Another study evaluated the cardioprotective functions of curcumin against oxidative stress and fibrosis. Diabetic mice with high dietary fat received A13 or curcumin through a nasogastric tube. Despite the control group, rats receiving A13 displayed less myocardial injury, reduced ROS and antioxidative enzymes and activated Nrf2/ARE pathway. These results shed light on potential therapeutic targets against cardiomyopathy [89].

Activation of Nrf2 was associated with JNK2 gene knockout in the aorta of the group of mice that underwent curcumin analog C66treatment. Histopathologic evaluations supportedC66 vascular defend against diabetes-induced vasculopathy [90].

#### 3.2.7. The Mechanism of HO-1 Pathway in Inflammation

A recent study was carried out to determine whether curcumin can activate the HO-1 pathway in vivo and in vitro in curcumin-mediated vascular conservation. After the induction of acute vasculitis in rabbits, the group that received curcumin for one month demonstrated lower inflammatory mediators and improved levels of Interstitial and blood levels of HO-1. It was found that the anti-inflammatory effects of curcumin were inhibited in the absence or inhibition of HO-1. Moreover, during in vitro assessments, they found that Nrf2 and p38 MAPK signaling pathways played acentral role in activating HO-1 in cultured human endothelium [91].

Two curcumin analogs 8d and 14p, displayed potent antioxidative impacts on H9c2 cells that underwent H_2_O_2_/TBHP induced oxidative stress. In vivo, 14p application decreased H_2_O_2_, TBHP-induced apoptosis andBax/Bcl-2–caspase-3 signaling while elevated MDA and SOD. Reducing myocardial infarction and apoptosis proved that 14p can attenuate reperfusion injury due to elevated intracellular ROS. However, curcumin failed to display antioxidative effects in the settings of Nrf2 knockout [92].

Mercury is well-known for its cytotoxic impact on human tissues. Zhao et al. assessed curcumin defense mechanisms in mice that underwent HgCl2 induced splenic injury. In the control group, phosphatidylinositol 3-kinase (PI3K)-AKT upregulation elevated the expression of genes involved in apoptosis and cellular injuries such as PI3K, AKT, LC3-II and p62. Nevertheless, curcumin attenuated increased intracellular sodium and extracellular calcium, resulting in spleen cell death by promoting the Nrf2 pathway [93]. Curcumin’s antioxidative effects on bladder function are dependent on the promotion of the Nrf2 HO-1 pathway. It limited apoptotic enzymes, supported NGF protein synthesis and applied neurorestorative impacts on the urinary bladder in cisplatin-induced bladder injury in rats [94].

To evaluate the effects of curcumin on necrotizing enterocolitis (NEC), newborn rats exposed to hypoxia and hypothermia received various curcumin dosages. According to the findings, increased SIRT1/NRF2 expression and hindered TLR4 and pyroptotic pathways can support anti-inflammatory features of curcumin [95]. Fattah et al. investigated cytoprotective capacities of resveratrol and curcumin in testicular dysfunction by di-(2-ethylhexyl) phthalate (DEHP) induced oxidative injury in rats. DEHP interfered with total antioxidant capacity (TAC) and metabolic actions of (GSH) and malondialdehyde (MDA) l. It also significantly reduced testosterone, acid and alkaline phosphatases (ACP and ALP), and lactate dehydrogenase (LDH). DEHP enhanced expression of testicular genes, including Nrf2, HO-1, HSP60, HSP70 and HSP90, along with inhibited c-Kit function. All destructive effects of this oxidant were considerably prevented by curcumin mainly due to promoting the Nrf2 signaling pathway [96].

## 4. Curcumin Performs Its Neuroprotective Roles viaNrf2

Worldwide one of the high mortality and morbidity public health problems is traumatic brain injuries. Unfortunately, there is no known effective treatment for Traumatic brain injuries. Despite the unclear curcumin mechanism of action, its benefits on neuroprotection were undeniable in vivo and in vitro. A study determined whether the neuroprotective role of curcumin in mouse TBI is dependent on the NF-E2-related factor (Nrf2) pathway. In this study, The Feeney weight-drop contusion model was used to mimic TBI. After 15 min of TBI induction, curcumin in the peritoneum was administered and at 24 h after TBI, brains were accumulated. At 24 h after TBI by Western blot and qRT-PCR, Scientists detected the levels of Nrf2 and its downstream genes including, Hmox-1, Nqo1, Gclm and Gclc. In addition, they evaluated edema, oxidative damage, cell apoptosis and inflammatory reactions, hoping to increase TBI treatment with curcumin. In wild-type mice, this study demonstrated, curcumin treatment was reduced bilateral cortex injury, neutrophil infiltration and microglia activation, improving neuron survival against TBI-induced apoptosis and degeneration. These effects were accompanied by the increased expression and nuclear translocation of Nrf2, and enhanced expression of antioxidant enzymes was the cause of the effects mentioned above. Their findings obtained that curcumin could activate the Nrf2 pathway. Therefore, the science presented a novel perspective for possible therapeutic use of curcumin for TBI and neuroprotective effects of Nrf2 [97,98,99].

Anti-inflammatory and antioxidative properties of curcumin protect the central nervous system from various brain-related diseases, including cerebral ischemia, intracerebral hemorrhage and Alzheimer’s disease. Scientists found that curcumin induced the nuclear translocation of Nrf2 in microglia, brain macrophage, therefore upregulated genes, such as heme oxygenase 1, NAD(P)H:quinone oxidoreductase 1, glutamate-cysteine ligase modifier subunit and ferritin light chain, and simultaneously downregulated lipopolysaccharide-induced inducible nitric oxide synthase expression, decreased antioxidant response on the human brain. In this study, scientists showed that curcumin’s anti-inflammatory effect in microglia is connected with its antioxidative effect in that CUR promotes Nrf2–p300 binding at the expense of p65–p300 binding. Since curcumin is a common spice that is used daily worldwide, it seems that curcumin could be therapeutically up or down-regulated genes to induce antioxidative effects and simultaneously ameliorate inflammatory conditions [100].

An abrupt increase in NAD(P)H andNQO1 antioxidative expression in ischemic stroke was prevented by post-treatment curcumin application. In Sprague–Dawley rats, ischemia due to middle cerebral obstruction was controlled by induction of Akt/Nrf2 and Nrf2/HO-1 signaling pathway [101,102]. Dietary curcumin considerably reduced the side effects of 4 graycarbon ion (^12^C^6+)^ cerebral radiation and improved memory loss by enhancing SOD and MDA. Activating HO-1, NQO and γ-glutamyl cysteine synthetase (γ-GCS) as downstream genes expressed in the Nrf2 pathway in curcumin-treated animal studies minimized detrimental effects of ^12^C^6+^ radiation on brain [103].

In the development of neurodegenerative disorders, such as Alzheimer’s disease (AD) and Parkinson’s disease (PD), oxidative stress and neuroinflammation are found to play a fundamental role. Nowadays, due to the diseases’ multifactorial characters, no effective therapies are available, thus underlying new strategies. There was a decrease in antioxidant defensive effects due to overexpression of the enzyme GSK-3β and downregulation of the Nrf2/ARE pathway. These pieces of evidence conclude the usefulness of dual GSK-3β inhibitors/Nrf2 inducers. In this regard, to design a dual modulator, the structures of a curcumin-based analog, as GSK-3β inhibitor and a diethyl fumarate fragment, as Nrf2 inducer, were combined. Amongst the hybrids, 5 and 6 proved to inhibit GSK-3β constructively, while 4 and 5 were remarkable activators of Nrf2 to increase the neuronal resistance of oxidative stress. These last pieces of evidence translated into specific neuroprotective effects of 4 and 5 against PD pathological events, including neurotoxicity elicited by α-synuclein aggregates and 6-hydroxydopamine. In addition, hybrid5 proved neuroprotective effects in a C. elegans model of PD where the activation of GSK-3β is intimately involved in Nrf2 regulation. For the final summary, five transpired as a fascinating multitarget derivative, worthy of being extracted in a multitarget PD perspective [104].

Transcription factor EB (TFEB) nuclear translocation is involved in the process of cellular autophagy, one of the significant homeostasis regulators. ROS plays a substantial role in the pathogenesis of degenerative neuronal disorders. It is speculated that cascades involved in TFEB transportation such as phosphorylation are restricted by curcumin. Song et al. found that this capability is attributed to the effects of this antioxidant on inhibiting glycogen synthase kinase-3β (GSK-3β) and upregulating Nrf2/HO-1 pathway in neuroblasts. Moreover, curcumin promoted the activation of TFEB-autophagy/lysosomal cascade. Therefore, it led to the degeneration of proteins associated with amyloidosis, such as amyloid-β precursor protein and –synuclein [105].

Diffuse axonal injury (DIA) resulted from brain trauma carries a dismal prognosis. Intraneuronal production of β-APP and p-tau proteins elevates the ROS load within the endoplasmic reticulum. Curcumin defended rat neuronal tissue from DIA-induced oxidative injury through enhancing Nrf2 nuclear translocation.

It also inhibited the intracellular accumulation of β-APP and p-tau proteins, attenuating neuronal injury and apoptosis. The curcumin-activated PERK/Nrf2 pathway induced homeostatic factors such as ATF4 while prevented CHOP signaling from promoting oxidative stress and apoptosis [106].

MeHg caused cellular malformations, elevated ROS and LDH, and subsequent increase in GSH and catalase (CAT) enzymes in astrocytes. Curcumin relieved astrocytes from methylmercury (MeHg) induced neurotoxicity and the consequent apoptosis in a time and dose-dependent pattern in rats. Furthermore, it promoted Nrf2 the following HO-1 and NADPH quinone reductase-1 (NQO1). Despite inhibitory effects of siRNA, curcumin’s function as Nrf2 activator was not adversely affected by pan-protein kinase C (PKC) inhibitor, Ro 31–8220 and the selective PKCδ inhibitor rottlerin [107].

Intrastriatal curcumin injection improved quinolinic acid (QUIN) induced neuronal impairment in Wistar rats. Curcumin prevented skeletal muscle weakness and structural malformations and maintained BDNF, ERK1/2 and Nrf2 within neurons. Enzymes involved in oxidation–reduction such as SOD, CAT and glutamylcysteine ligase (-GCL) were upregulated in rats that received curcumin. Furthermore, brain-derived neurotropic factor (BDNF) stimulated the mitogen-activated protein kinase (MAPK) protein extracellular signal-regulated kinase-1/2 (ERK1/2) that promoted antioxidative features of Nrf2 in the curcumin group [108].

Substantia nigra pars compacta (SNpc), where dopaminergic neurons are located, undergoes degeneration in Parkinson’s disease (PD). The destructive impacts of ROS have been proposed to play a crucial role in the pathogenesis of PD. In rats that underwent SNpc damage by rotenone, curcumin alleviated oxidative stress and subsequently improved motor function. These rats demonstrated higher levels of GSH, HO-1,NAD(P)Quinone oxidoreductase-1, reduced ROS and malondialdehyde levels which was attributed to curcumin-induced Akt/Nrf2 activation [109].

The oxidized heme called hemin, impaired cerebellar granule neurons (CGNs) of rats. However, curcumin administration significantly prevented apoptosis by decreasing ROS and GSH/glutathione disulfide (GSSG) ratio. Curcumin antioxidative functions in producing glutathione reductase, glutathione S-transferase and superoxide dismutase highly depended on Nrf2 induced HO-1 and GSH production [110].

Ischemic stroke-induced reperfusion injury exposes neurons to considerable ROS and inflammation. Curcumin considerably prevented neurotoxicity in Wistar rats with reperfusion brain damage by blockage of a middle cerebral artery. Enhancing Nrf2 and inhibiting NF-κB in the curcumin group protected neuronal tissue against edema and the following necrosis in Wistar rats [111,112].

Inflammation and oxidative stress play a crucial role in destroying the white matter of the central nervous system. Hypoxic microenvironment increased the expression of HIF1a in Sprague Dawley rats with absent spinal cord white matter. Curcumin promoted glial fibrillary acidic protein (GFAP) and neurofilament-H (NF-H) expression while decreased the expression of HIF1a produced in hypoxic settings. Furthermore, it down-regulated TNF-α and IL-1 causing cellular death by activating Nrf2 signaling pathway [113].

## 5. Other Cross Talks between Curcumin and Nrf2

An amoebic liver abscess (ALA) is one of the manifestations of extraluminal amoebiasis. Curcumin has been proposed to have anti-amoebic properties; it protected hepatocytes against *Entamoeba histolytica* in hamsters. Aside from low ALT, ALP and γ-GTP serum levels in curcumin-treated hamsters, it preserved the glycogen storage by limiting Entamoeba access. Curcumin decreased NF-κB and IL-1*β* expression while promoted Nrf2 pathway [114].

Gram-negative sepsis is one of the significant side effects of gastrointestinal infections with a high mortality rate. An intravenous curcumin analog named intracorporeal chitosan-coated curcumin nanocrystals (Chi-CURNC- 4b) promoted Nrf2 expression and deactivated NFκB which suppressed inflammatory mediators involved in LPS induced sepsis in mice [115].

ROS plays a significant role in the pathophysiology of chronic kidney disease (CKD). The association of curcumin with mycophenolate mofetil (MMF) in mice that underwent nephrectomy protected the kidney from oxidative stress by improving autoregulation, proteinuria and hypertension. The protective effects of this intervention are attributed to maintaining Nrf2-Keap1 pathway and avoiding its nuclear translocation [116].

Curcumin has shown antioxidative capacities, possibly by supporting phase II enzymes involved in redox reactions. Its dietary supplementation accompanied by sulforaphane (SFN) in HepG2-C8 cells induced the activation of Nrf2 and ARE-luciferase and up-regulated the expression of HO-1 and UGT1A1. Therefore, the combination of these two natural compounds may have antineoplastic impacts [117,118].

Liver fluke infection treatment of the anthelmintic praziquantel causes hepatotoxicity by induction of oxidative stress. Curcumin has shown antioxidative characteristics against praziquantel in hamsters infected by *Opisthorchis viverrini*. It reduced the eosinophilia associated with parasitic infections while stimulated interstitial lymphocytes aggregation.

Nrf2 and its connected pathways (Keap 1, NAD(P)H:quinine oxidoreductase 1, glutamate-cysteine ligase, activating transcription factor 3, peroxiredoxin 3, peroxiredoxin 6, manganese superoxide dismutase and catalase) are the crucial pathways through which curcumin boosts the antioxidant potential of serum ferric ion. Furthermore, curcumin reduced biomarkers that represent liver damage (urinary 8-oxo-7,8-dihydro-20-deoxyguanosine, serum malondialdehyde and nitrate and plasma ALT) and inhibited signaling pathways and mediators involved in inflammation [119].

Small intestinal injury happens due to the oxidative microenvironment in intrauterine growth restriction (IUGR). Studies that assessed the antioxidative impacts of dietary curcumin in IUGR pigs found that curcumin improved jejunal oxidative stress through activation of Nrf2, glutamate-cysteine ligase catalytic subunit (GCLC), superoxide dismutase 1 (SOD1), glutamate-cysteine ligase modifier subunit (GCLM) and NAD(P)H quinone dehydrogenase 1 (NQO1). In addition, curcumin inhibited inflammatory mediators and enzymes involved in apoptosis [120,121].

Heart failure patients cannot stand physical activity which might be attributed to dysfunctional Nrf2 expression in skeletal muscles. Curcumin induced Nrf2 pathway in heart failure mice, improved muscular strength and exercise tolerance by increasing threshold of muscle exhaustion [122,123]. Some studies evaluated the antioxidative impacts of dietary curcumin in the development and immunological function of grass carp by assessing the expression of NF-κB and Nrf2 pathways. Besides promoting antioxidative enzymes, curcumin significantly prevented muscular damage by inhibiting heat shock protein along with NF-κB and Nrf2 pathways, therefore decreasing muscular malondialdehyde and lactic acid [124,125].

Dietary curcumin enhanced growth and decreased mortality among juvenile grass carp.Lysozyme (LYZ), acid phosphatase (ACP) and complement 3 (C3) and C4 were activated whileALT and AST blood levels were reduced. Curcumin inserted its anti-inflammatory features by promoting mRNA levels of LYZ, C3 bactericidal or bacteriostatic peptides (hepcidin, liver-expressed antimicrobial peptide-2 (LEAP-2), β-defensin), anti-inflammatory mediators (interleukin-10 (IL-10) and transforming growth factor β1 (TGF-β1)) and κBα (IκBα) inhibitor. Moreover, curcumin inhibited inflammatory mediators and enhanced enzymes that are involved in oxidation-reduction [126].

## 6. Conclusions and Future Prospects

Curcumin is found to be effective in the treatment of cancer and other pathological conditions. Curcumin has shown a variety of potential bioactivities such as anti-inflammatory, antioxidant, anti-infective properties with few side effects. Curcumin has been proved as natural activators of Nrf2 pathway. Curcumin attenuated increased intracellular sodium and extracellular calcium, resulting in spleen cell death by promoting the Nrf2 pathway. The interconnected anti-inflammatory and antioxidative properties of curcumin resulted from its modulatory effects on Nrf2 signaling pathway which have been shown to improve insulin resistance. Curcumin exerts its anti-inflammatory impacts through suppressing metabolic reactions and proteins such as Keap1 that provoke inflammation and oxidation. Moreover, the role of inflammatory mediators like tumor necrosis alpha (TNF-α) in supporting Keap1 production and Nrf2 pathway inactivation is prevented mainly by this natural polyphenol.

The discovery of novel chemotherapeutic products emphasizes on medications with well-defined mechanisms of action, in order to boost anticancer effects while reducing toxicity to healthy tissue. The question of whether Nrf2 can be utilized as a pharmaceutical target must be evaluated against future clinical investigations and the inclusion of oxidative stress mediators in cancer chemotherapy. Therefore, the innovation, design and synthesis of Nrf2 centered approaches are significant and difficult challenges that could lead to innovative cancer therapeutic strategies. Curcumin’s effects on additional molecular targets, such as the Nrf2 and catenin pathways, have also been briefly reviewed. Though preliminary research in animals and cell lines has demonstrated curcumin’s chemopreventive properties, substantial proof from epidemiological research and clinical trial outcomes will be required to advance curcumin’s commercialization as a cancer preventative and therapeutic agent.

## Data Availability

Not applicable.

## Abbreviations

Nrf2	Nuclear factor erythroid 2 p45-related factor 2)
bZIP	Basic leucine zipper protein
ARE	antioxidant response elements
EpRE	Electrophile response elements
Keap1	Kelch-like ECH-associated protein 1
GLCM	glutamate-cysteine ligase
HO-1	heme oxygenase-1
PhIP	Amino-1-methyl-6-phenylimidazo[4,5-b]pyridine
BGS	bisulfite genomic sequencing
CAPE	caffeic acid phenethyl ester indirectly
H2O2	hydrogen peroxide
CAT	Catalase
SOD	superoxide dismutase
GSHPX	glutathione peroxidase
PEITC	phenethyl isothiocyanate
NAFLD	Non-alcoholic fatty liver disease
LDH	lactate dehydrogenase
QCT	Quinocetone
iNOS	nitric oxide synthase
ZEA	Zearalenone
